# BTN3A Targeting Vγ9Vδ2 T Cells Antimicrobial Activity Against *Coxiella burnetii*-Infected Cells

**DOI:** 10.3389/fimmu.2022.915244

**Published:** 2022-06-27

**Authors:** Laetitia Gay, Soraya Mezouar, Carla Cano, Etienne Foucher, Mélanie Gabriac, Marie Fullana, Loui Madakamutil, Jean-Louis Mège, Daniel Olive

**Affiliations:** ^1^Aix-Marseille University (Univ), IRD, Assistance Publique Hopitaux de Marseille (APHM), Microbe, Evolution, Phylogeny, Infection (MEPHI), Marseille, France; ^2^IHU-Méditerranée Infection, Marseille, France; ^3^ImCheck Therapeutics, Marseille, France; ^4^Aix-Marseille University (Univ), Assistance Publique Hopitaux de Marseille (APHM), Hôpital de la Conception, Laboratoire d’Immunologie, Marseille, France; ^5^Centre de Recheche contre le cancer de Marseille (CRCM), Inserm UMR1068, Centre national de la recherche scientifique (CNRS) UMR7258, Institut Paoli Calmettes, Marseille, France

**Keywords:** *Coxiella burnetii*, Vγ9Vδ2 T cells, butyrophilin, antimicrobial immunity, therapeutic approaches

## Abstract

Vγ9Vδ2 T cells have been reported to participate to the immune response against infectious diseases such as the Q fever caused by *Coxiella burnetii* infection. Indeed, the number and proportion of Vγ9Vδ2 T cells are increased during the acute phase of Q fever. Human Vγ9Vδ2 T cell responses are triggered by phosphoantigens (pAgs) produced by pathogens and malignant cells, that are sensed *via* the membrane receptors butyrophilin-3A1 (BTN3A1) and -2A1 (BTN2A1). Here, by using CRISPR-Cas9 inactivation in THP-1 cells, we show that BTN3A and BTN2A are required to Vγ9Vδ2 T cell response to *C. burnetii* infection, though not directly involved in the infection process. Furthermore, *C. burnetii*-infected monocytes display increased BTN3A and BTN2A expression and induce Vγ9Vδ2 T cell activation that can be inhibited by specific antagonist mAb. More importantly, we show that the antimicrobial functions of Vγ9Vδ2 T cells towards *C. burnetii* are enhanced in the presence of an BTN3A activating antibody. This supports the role of Vγ9Vδ2 T cells in the control of *C. burnetii* infection and argues in favor of targeting these cells as an alternative treatment strategy for infectious diseases caused by intracellular bacteria.

## Introduction

The role of Vγ9Vδ2 T cells in the host immune response to bacterial infection is now well-documented (1). Human Vγ9Vδ2 T cells, which normally represent 2-5% of peripheral blood T cells, are expanded in infected patients to reach up to ≥50% of the circulating T cells (2, 3), as reported for patients undergoing mycobacterial disease, listeriosis, salmonellosis, brucellosis, tularemia, legionellosis and Q fever (4–10). Furthermore, local expansion of Vγ9Vδ2 T cells have also been reported in the bronchoalveolar lavage fluids from patients with active pulmonary tuberculosis and in cerebral spinal fluids from patients with bacterial meningitis (11–13). Two direct antimicrobial actions of Vγ9Vδ2 T cells against various viruses, protozoa and bacteria were reported, including cytotoxic activity to pathogen-infected cells and a cell-mediated non-cytolytic activity based on cytokine production (1, 14–16). *In vitro* studies have shown that Vγ9Vδ2 T cells are able to effectively kill intracellular pathogens such as *M. tuberculosis, L. monocytogenes*, and *Brucella suis* (17–21).

The butyrophilin 3A1 (BTN3A1) cell surface molecule is involved in cell recognition and the human Vγ9Vδ2 T cells activation (22, 23). Vγ9Vδ2 T cells are activated by small, phosphorylated nonpeptide antigens, called phosphoantigens (pAgs) (14). The production of these metabolites is increased in tumor or stressed eukaryotic cells, and can be naturally produced by several pathogens (11, 24, 25). Among the *BTN3A* isoforms (*BTN3A1*, *BTN3A2*, *BTN3A3*), *BTN3A1* is unique in that its intracellular B30.2 domain binds to pAgs (26, 27), while its juxtamembrane domain performs a critical function in homodimerization and heterodimerization of BTN3A (28). Conformational changes in the juxta-membrane domain, induced by the binding of pAgs to the B30.2 domain, are involved in Vγ9Vδ2 T cell activation (29). More recently, BTN2A1 has been identified as a novel actor in pAg sensing by Vγ9Vδ2 T cells (30–32). BTN2A1 is a direct ligand for the Vγ9 TCR interacting with BTN3A1 to trigger Vγ9Vδ2 TCR activation (30).

Several evidences highlight the key role of Vγ9Vδ2 T cells in Q fever, an infectious disease caused by the intracellular bacterium *Coxiella burnetii*. (**1**) During the acute phase of the disease, the numbers and proportion of Vγ9Vδ2 T cells were found increased (**2**) with a significant increase of the expression of HLA-DR, but not CD25 (10). In this study, we investigated the functional role of Vγ9Vδ2 T cells and the involvement of BTN3A and BTN2A in host defense against *C. burnetii*. Here, we observed that *C. burnetii* infection of healthy monocytes lead to the increase of the expression of these two BTNs. Using a CRISPR-Cas9 knockout model in the THP-1 cell line, we observed that BTN3A and BTN2A are not directly involved in the infection process by *C. burnetii* but play a role in the host immune response to infection. We reported that infected monocytes induced Vγ9Vδ2 T cell activation in a BTN3A and BTN2A dependent manner. Finally, the use of a BTN3A activating antibody enhances the antimicrobial functions of Vγ9Vδ2 T cells against *C. burnetii* infected cells through the production of cytotoxic molecules and large amounts of IFN-γ and TFN-α. Our results highlight the role of Vγ9Vδ2 T cells in the control of *C. burnetii* infection and the therapeutic potential of BTN3A activating antibody in infections.

## Materials and Methods

### Cell Isolation

Blood samples (leucopacks) were obtained from the local French Blood Establishment (*Etablissement français du sang*, EFS), which carries out donor inclusions, informed consent, and sample collection. Through a convention established between our laboratory and the EFS (N°7828), buffy coats were obtained and peripheral blood mononuclear cells (PBMCs) were isolated as previously described (33). Monocytes were purified from PBMCs using anti-CD14-conjugated magnetic beads (Miltenyi Biotec, Bergisch Glabach, Germany) and cultured in Roswell Park Memorial Institute-1640 medium (RPMI, Life Technologies, Carlsbad, CA, USA) containing 10% fetal bovine serum (FBS, Gibco, Life technologies), 2 mM L-glutamine, 100 U/mL penicillin and 50 µg/mL streptomycin (Life Technologies).

Vγ9Vδ2 T cells were expanded from fresh PBMCs as previously described (34, 35). Briefly, PBMCs were cultured in RPMI-1640 medium supplemented with 10% FBS, interleukin-2 (IL-2, 200 UI/ml) and Zoledronic acid monohydrate (to a final concentration of 1 µM). IL-2 was added every 2 days beginning on day 5 for 12 days and the purity of the Vγ9Vδ2 T cells was assessed by flow cytometry analysis (>85%) and then frozen at -80°C in 10% dimethyl sulfoxide (Sigma-Aldrich, Saint-Quentin-Fallavier, France) and 90% FBS.

### Lentiviral *Transduction* and CRISPR-Cas9-*Mediated* BTN3A or BTN2A *Knockout*

For all transductions, THP-1 cells were seeded in 12-wells plates (2.5x10^5^ cells/well), and 25 μL of concentrated lentiviral particles were added to the culture. After 24 hours, cells were washed twice in complete medium, and cultured in their regular culture medium for 48 hours. Optimized CRISPR target sequences targeting the three *BTN3A* gene isoforms and for BTN2A gene inactivation, targeting both *BTN2A* gene isoforms (sequence available upon request) were cloned into the lentiCRISPR-v2 vector (Addgene #52961). For selection of THP-1 transductants, 1 μg/mL puromycin was added to the culture medium (**Supplementary Figure 1**).

### Bacterial Production

*Coxiella burnetii* phase I (Nine Mile (NM) strain, RSA493 and Guiana strain, MST17) were cultured in L929 cells for 10 days, as previously described (36). Briefly, infected cells were sonicated and centrifuged at 10,000*g* for 10 minutes, then washed and stored at -80°C. Bacterial titers were determined using Gimenez staining, and bacterial viability was assessed using the Live/Dead BacLight bacterial viability kit (Molecular Probes, Eugene, OR, USA).

*Mycobacterium tuberculosis* (H37Rv strain) was cultured in Middelbrook 7H10 (Becton Dickinson, Le Pont de Claix, France) supplemented with 10% oleic acid-albumin-dextrosecatalase (OADC, Becton Dickinson), as previously described (37). Prior to infection, the colonies were resuspended in phosphate buffered saline (PBS, Life Technologies), vigorously vortexed for 10 min using 3 mm sterile glass beads (Sigma-Aldrich) and passed 10 times through a 25 G needle to disperse clustered cells. Calibration was performed at OD 580 nm and confirmed by counting mycobacteria after Ziehl-Neelsen staining.

### Cell Infection

Monocytes isolated from healthy donors were infected with *C. burnetii* strains (50 MOI) or with *M. tuberculosis* (5 MOI). After 24 hours of infection, the expression of BTN3A and BTN2A were investigated by qRT-PCR and flow cytometry. For co-cultures experiments, monocytes isolated from healthy donors previously infected 24 hours with *C. burnetii* strains or with *M. tuberculosis* were co-cultured with autologous Vγ9Vδ2 T cells (E:T ratio of 1:1). After 4 hours of co-culture, Vγ9Vδ2 T cell degranulation and cytotoxicity was assessed by flow cytometry and the bacterial load was measured by flow cytometry and qPCR. Finally, the supernatants of the co-cultures were analyzed for the presence of cytokines and cytotoxic molecules by ELISA assay.

### Bacterial Detection

DNA was extracted from *C. burnetii* infected cells using a DNA Mini Kit (Qiagen, Courtaboeuf, France). Bacterial load was quantified using real time quantitative PCR (qPCR) performed with specific primers F (5’-GCACTATTTTTAGCCG-GAACCTT-3’) and R (5’-TTGAGGAGAAAAACTGGATTGAGA-3’) targeting the *C. burnetii COM-1* gene, as previously described (36).

The presence of *C. burnetii* within cells was also assessed by flow cytometry. Briefly, infected cells were fixed with 4% paraformaldehyde and permeabilized with 0.1% Triton X-100 (Sigma-Aldrich). After washing, cells were incubated with a rabbit antibody directed against *C. burnetii* for 30 min and then with an Alexa 647 anti-rabbit antibody (Invitrogen). Data were collected on a BD Canto II instrument (BD Biosciences, Le Pont-de-Claix, France) and analyzed with FlowJo software (FlowJo v10.6.2, Ashland, OR).

For *M. tuberculosis* infected cells, DNA was extracted from infected cells as follows: aliquots of 150 μL were incubated overnight at 56°C with 150 μL of G2 buffer mixed with 15 μL proteinase K (20 mg/mL). After two cycles of mechanical lysis (45 s), the total DNA was extracted using the EZ1 DNA Tissue Kit (Qiagen). *M. tuberculosis* DNA detection was performed targeting the *M*. *tuberculosis* internal transcribed spacer (ITS) (**Table 1**), as previously described (37).

**Table 1:** Primers used for the response to infection.

Gene	Forward primer (5’-3’)	Reverse primer (5’-3’)	
*ACTB*	GGAAATCGTGCGTGACATTA	AGGAGGAAGGCTGGAAGAG	
*GAPDH*	Hs02786624_g1
**M1 genes**			
*TNF*	AGGAGAAGAGGCTGAGGAACAAG	GAGGGAGAGAAGCAACTACAGACC	
*IL1B*	CAGCACCTCTCAAGCAGAAAAC	GTTGGGCATTGGTGTAGACAAC	
*IL6*	CCAGGAGAAGATTCCAAAGATG	GGAAGGTTCAGGTTGTTTTCTG	
*IFNG*	GTTTTGGGTTCTCTTGGCTGTTA	ACACTCTTTTGGATGCTCTGGTC	
*CXCL10*	TCCCATCTTCCAAGGGTACTAA	GGTAGCCACTGAAAGAATTTGG	
**M2 genes**			
*IL10*	GGGGGTTGAGGTATCAGAGGTAA	GCTCCAAGAGAAAGGCATCTACA	
*TGFB*	GACATCAAAAGATAACCACTC	TCTATGACAAGTTCAAGCAGA	
*IL1RA*	TCTATCACCAGACTTGACACA	CCTAATCACTCTCCTCCTCTTCC	
*CD163*	CGGTCTCTGTGATTTGTAACCAG	TACTATGCTTTCCCCATCCATC	
**BTN isoform genes**			
*BTN3A1*	TTCCAGGTCATAGTGTCTGC	TGAGCAGCTGAGCAAAAGG	
*BTN3A2*	TGGGAATACCAAGGGA	AGTGAGCAGCTGGACCAAGA	
*BTN3A3*	GAGGGAATACTAAGAAATGGT	GAAGAGGGAGACATGAAAGT	
*BTN2A1*	Hs00924832_m1
*BTN2A2*	Hs00950165_g1
***C. burnetii* gene**			
*CB COM-1*	GCACTATTTTTAGCCG-GAACCTT	TTGAGGAGAAAAACTGGATTGAGA	
MTB *ITS*	CAAGGCATCCACCATGCGC	GGGTGGGGTGTGGTGTTTGA	

### RNA Isolation and q-RTPCR

Total RNA was extracted from infected cells (2x10^6^ cells/well) using the RNeasy Mini Kit (Qiagen) with DNAse I treatment as previously described (38). RNAs quality and quantity were evaluated using a NanoDrop spectrophotometer (Nanodrop Technologies, Wilmington, USA). Reverse transcription was performed using M-MLV Reverse Transcriptase kit (Life Technologies) and oligo(dT) primers. The expression of genes characteristics of M1/M2 macrophage phenotypes, as well as *BTN3A* isoform genes, was evaluated using real time qPCR, Smart SYBR Green fast Master kit (Roche Diagnostics, Meylan, France) and specific primers (**Table 1**). *BTN2A* levels expression was evaluated using real time qPCR, TaqMan^®^ Fast Advanced Master Mix (Applied Biosystems, Life Technologies) and specific probes (**Table 1**). All qPCRs were performed using a CFX Touch Real-Time PCR Detection System (Bio-Rad, Marnes-la-Coquette, France). Results were normalized by the expression of *ACTB* or *GAPDH* housekeeping gene and are expressed as relative expression of investigated genes with 2^-ΔCt^ where ΔCt = Ct_target_ – Ct_housekeeping gene_ as previously described (36).

### BTN3A and BTN2A Surface Expression

Cells were suspended in PBS (Life Technologies) containing 1% FBS and 2 mM EDTA (Sigma-Aldrich). Cells were labeled with viability dye (Live/Dead Near IR, Invitrogen), mouse anti-BTN3A (clone 103.2) or anti-BTN2A (clone 7.48) Abs or with the appropriate isotype control (Miltenyi Biotech). After 30 min incubation, primary antibody binding was detected with secondary PE anti-mouse antibody (Invitrogen) and data were collected on a Navios instrument (Beckman Coulter) and analyzed with FlowJo software (FlowJo v10.6.2).

### Degranulation Assay

Monocytes were co-cultured with Vγ9Vδ2 T cells at effector-to-target (E:T) ratio of 1:1 in presence of mouse anti-BTN2A mAb (clone 7.48) or mouse anti-BTN3A mAb (clones 20.1 or 103.2) and fluorochrome-labeled CD107a and CD107b (BD Biosciences). Phorbol 12-myristate 13-acetate (PMA, 20 ng/mL) with ionomycine (1 µg/mL) were used as positive control for Vγ9Vδ2 T cell activation. After 4 hours, cells were harvested and stained with fluorochrome-labeled TCR-specific mAbs (Miltenyi Biotec) and a viability marker (Live/Dead Near IR, Invitrogen). The degranulation was evaluated by flow cytometry as the percentage CD107a/b^+^ cells in the γδ T cell population (**Supplementary Figure 2**). Data were collected on a Navios instrument (Beckman Coulter) and analyzed with FlowJo software (FlowJo v10.6.2).

### Cytotoxicity Assay

Monocytes were labeled with 10 µM Cell Proliferation Dye eFluor^®^ 670 (Invitrogen) and then co-cultured with Vγ9Vδ2 T cells at E:T ratio of 1:1 in presence of mouse anti-BTN3A mAb (clone 20.1) at the indicated concentrations. After 4 hours, cells were stained with CellEvent Caspase-3/7 Green (Invitrogen) to identify dead cells. The cytotoxicity was assessed by flow cytometry as the percentage of Caspase 3/7^+^ cells in the target cell population (**Supplementary Figure 2**). Data were collected on a BD Canto II instrument (BD Biosciences) and analyzed with FlowJo software (FlowJo v10.6.2).

### Immunoassays

Tumor necrosis factor-alpha (TNFα), interferon*-*gamma (IFNγ), Granulocyte-macrophage colony-stimulating factor (GM-CSF) (R&D Systems), granzyme B, perforin, and granulysin (Abcam) levels were quantified in the supernatants of monocyte/Vγ9Vδ2 T cells co-cultures using specific immunoassay kits. TNFα, IFNγ, interleukin (IL)-1β, IL-6, IL-10 and transforming growth factor beta (TGF-β) (R&D Systems) levels were quantified in the supernatants of BTNs KO cells following *C. burnetii* infection. The sensitivity of assays was 6.2 pg/mL for TNFα, 5.7 pg/mL for IFNγ, 1.0 pg/mL for IL-1β, 0.7 pg/mL for IL-6, 3.9 pg/mL for IL-10, 15.4 pg/mL for TGF-β, 3.0 pg/mL for GM-CSF, 20 pg/mL for granzyme B, 40 pg/mL for perforin and 10 pg/mL for granulysin.

### Statistical Analysis

Statistical analysis was performed with GraphPad Prism (8.0, La Jolla, CA). After analysis of the distribution of the data with a normality test, the Mann-Whitney *U* test was used as a non-parametric test and the *t* test as a parametric test. Hierarchical clustering of gene expression was analyzed using the ClustVis webtool (39). The limit of significance was set up at *p<0.05*.

## Results

### *C. burnetii* Infection Enhances Expression of BTN3A and BTN2A

**Figure 1:**
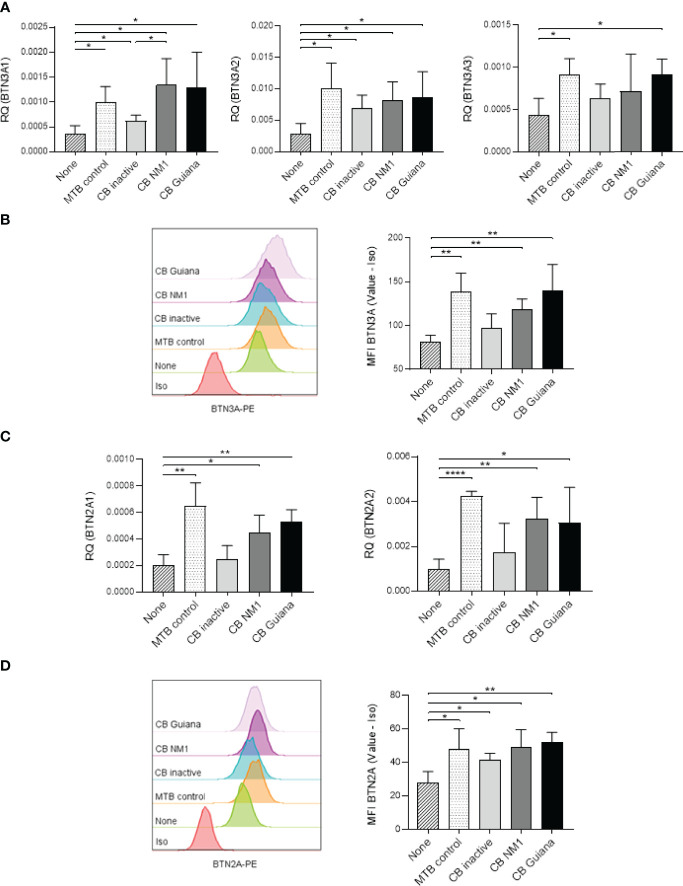
Bacterial infections modulate BTN3A and BTN2A expression. Monocytes isolated from healthy donors (n = 4) were infected with *C. burnetii* strains (50 MOI) or with *M. tuberculosis* (5 MOI) for 24 hours. **(A)** The relative gene expression of *BTN3A* isoforms (A1, A2, A3) and **(B)** the BTN3A protein expression were investigated by qRT-PCR and flow cytometry, respectively. **(C)** The relative gene expression of *BTN2A* isoforms (A1, A2) and **(D)** the BTN2A protein expression were investigated by qRT-PCR and flow cytometry, respectively. Data were analyzed using a normality test and a parametric *t* test. Values represent mean ± standard deviation. *p < 0.05, **p < 0.01, and ****p < 0.0001.

To assess whether *C. burnetii* infection affected the expression of BTNs, monocytes from healthy donors were isolated and infected with the reference strain NM1 or with the Guiana strain, described to be more virulent (40, 41). After 24 hours of incubation with active or heat-inactivated *C. burnetii* NM1 strain, increases of transcript expression of both *BTN3A1* and *BTN3A2* isoforms, but not of *BTN3A3* were found. Guiana strain infection enhanced the expression of all three isoforms, similar to *M. tuberculosis* infection used as control (**Figure 1A**) (23). Interestingly, significant differences of *BTN3A1* expression were observed between cells infected with active or heat-inactivated *C. burnetii* NM1 (p=0.0374), suggesting that virulence affected *BTN3A1* expression. Indeed, inactivated form of *C. burnetii* are reported to induce a weaker modulation of the expression of the A1 isoform, the essential form for pAg-mediated activation of Vγ9Vδ2 T cells (23). Significant increase of BTN3A protein expression was found for monocytes infected with *C. burnetii* NM1 and Guiana strains (p=0.0021 and p=0.0096, respectively) (**Figure 1B**).

As BTN2A is involved in Vγ9Vδ2 T-cell activation (31), we also investigated whether *C. burnetii* infection affected its expression. After 24 hours of infection, *BTN2A* transcriptional expression for both isoforms (BTN2A1 and BTN2A2) was significantly increased after *C. burnetii* NM1 and Guiana infection (BTN2A1 p=0.0170 and p=0.0021, respectively; and BTN2A2 p=0.0054 and p=0.0463, respectively) compared to uninfected cells and without significant modulation compared to the heat-inactivated form (**Figure 1C**). Regarding BTN2A protein expression, a significant increase was observed for *C. burnetii* infected monocytes (NM1 strain, p=0.0160; and Guiana strain, p=0.0018) compared to uninfected cells, as observed for *M. tuberculosis* infection as control (**Figure 1D**).

Altogether, like *M. tuberculosis* infection, *C. burnetii* infection leads to increased expression of BTN3A and BTN2A in infected cells.

### Involvement of BTN3A and BTN2A in *C. burnetii* Infection

**Figure 2:**
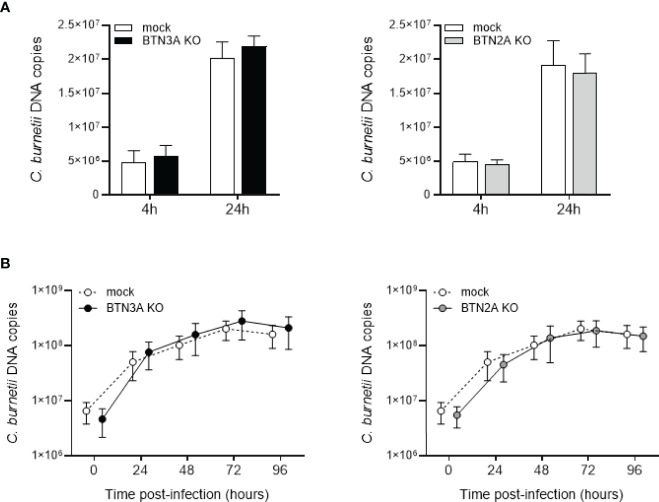
Involvement of BTN3A and BTN2A in *C. burnetii* infection. CRISPR-Cas9-mediated inactivation of BTN3A or BTN2A was performed in THP-1 cell line. THP-1 cells transduced with a guide targeting all *BTN3A* isoforms (BTN3KO) or all *BTN2A* isoforms (BTN2AKO) or with an irrelevant CRISPR guide (mock) for control cells were infected with *C. burnetii* NM1 (50 MOI) (n = 3). **(A)** After 4 and 24 hours of infection, the number of bacterial DNA copies within THP-1 cells was assessed by qPCR. **(B)** THP-1 cells were incubated with *C. burnetii* for 4 h (day 0), then washed to eliminate free bacteria and incubated for 4 days. Each day, the number of bacterial DNA copies was evaluated by qPCR. Values represent mean ± standard deviation.

Next, we investigated whether BTNs could be involved in the uptake or replication of *C. burnetii*. For this purpose, we performed a CRISPR-Cas9 knockout of the three *BTN3A* genes or the two *BTN2A* genes in the THP-1 cell line. Cells were transduced with a guide targeting either *BTN2A1* and *2A2* (BTN2AKO) or *BTN3A1, 3A2* and *3A3* (BTN3AKO) isoforms or with an irrelevant CRISPR guide (mock). BTN3AKO, BTN2AKO and mock cells were infected with *C. burnetii* NM1, and the bacterial load was assessed by qPCR. No differences were observed concerning the bacterial load (**Figure 2A**) and replication overtime (**Figure 2B**) between BTN3AKO, BTN2AKO and mock cells, suggesting that BTN3A and BTN2A are not directly involved in the process of *C. burnetii* infection.

### Involvement of BTN3A and BTN2A in the Inflammatory Response to *C. burnetii* Infection

**Figure 3:**
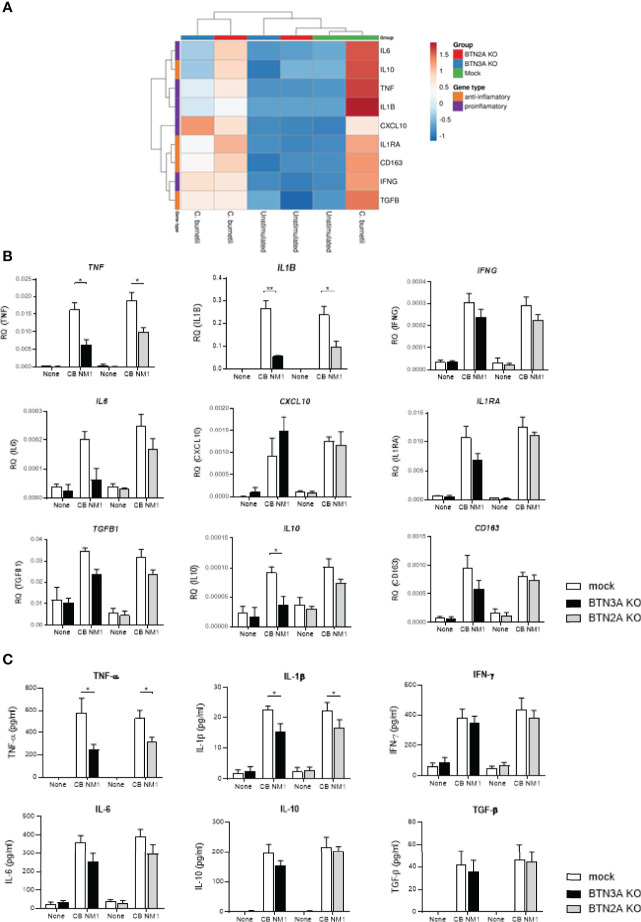
Involvement of BTN3A and BTN2A in the inflammatory response to *C. burnetii* infection. THP-1 cells transduced with an irrelevant CRISPR guide (mock) or a guide targeting all *BTN2A* isoforms (BTN2AKO) or all *BTN3A* isoforms (BTN3AKO) were infected with *C. burnetii* NM1 (100 MOI) (n = 3). After 24 hours infection, the expression of genes involved in the inflammatory (*TNF*, *IL1B*, *IL6*, *IFNG*, *CXCL10*) or immunoregulatory (*IL10*, *TGFB1*, *IL1RA*, *CD163*) response was investigated by quantitative reverse-transcription polymerase chain reaction after normalization with housekeeping actin gene as endogenous control. Data are illustrated as **(A)** hierarchical clustering obtained using ClustVis webtool or **(B)** relative quantity of investigated genes. **(C)** After 24 hours infection, TNF-α, IL-1β, IFN-γ, IL-6, IL-10, and TGF-β release were evaluated in the culture supernatants by ELISA assay. Data were analyzed using a normality test and a parametric *t* test. Values represent mean ± standard error. *p < 0.05 and **p < 0.01.

We then investigated the involvement of BTNs in the host immune response following *C. burnetii* infection in THP-1 cells. As observed in the **Figure 3**, *C. burnetii* infection results in modulation of genes characteristics of both pro-inflammatory (*TNF, IFNG, IL6, CXCL10, IL1RA* and *IL1B*) and anti-inflammatory (*IL10, TGFVB*and *CD163*) responses in THP-1 cells. Upon infection with *C. burnetii*, the hierarchical clustering based on the expression of the above mentioned genes revealed that BTNs expression correlated with the transcriptional response to infection, as depicted by a separate clustering of BTN3AKO/BTN2AKO cells and mock cells (**Figure 3A**). Indeed, BTN3AKO and BTN2AKO cells displayed significantly decreased expression of inflammatory genes following *C. burnetii* infection, in particular that of *TNF* and *IL1B* (**Figure 3B**). Also, *IL6* transcriptional expression appear to be affected by the BTN3A KO (p=0.0862) but not by the BTN2A KO. Furthermore, the expression of *IL10* transcript was significantly decreased compared to mock cells (p=0.0435) (**Figure 3B**). Consistently, BTN3AKO and BTN2AKO cells presented a significant decrease in TNF and IL-1β release following *C. burnetii* infection compared to mock cells (**Figure 3C**). No significant difference in the levels of anti-inflammatory cytokines such as IL-10 and TGF-β was observed.

Taken together, these data reported that both BTN3A and BTN2A are involved in the inflammatory response to *C. burnetii* infection.

### *C. burnetii* Infection Leads to Vγ9Vδ2 Cells Activation in a BTN3A and BTN2-Dependent Manner

**Figure 4:**
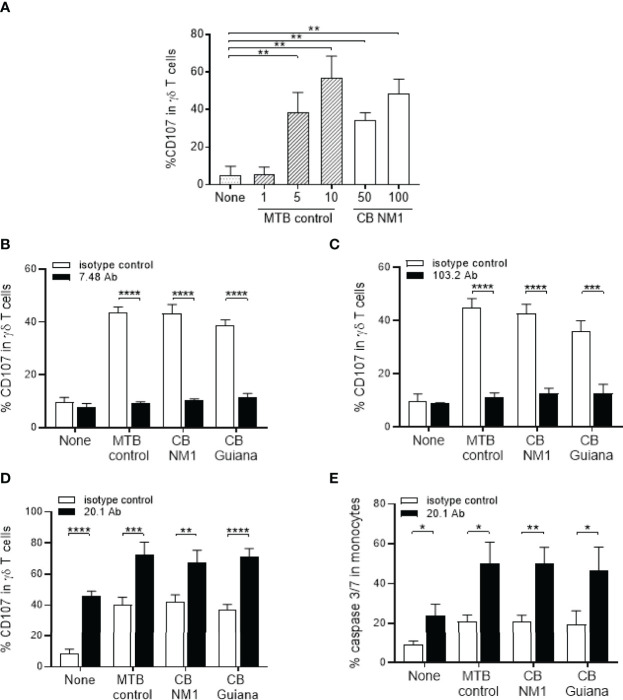
Infection with *C. burnetii* leads BTN3A and BTN2-dependent activation of Vγ9Vδ2 T lymphocytes. **(A)** Monocytes isolated from healthy donors (n = 3) previously infected 24 hours with *C. burnetii* NM1 (50 or 100 MOI) or with *M. tuberculosis* (1, 5 or 10 MOI) were co-cultured with autologous Vγ9Vδ2 T cells (E:T ratio of 1:1). Vγ9Vδ2 T cell degranulation (%CD107ab+ cells) was assessed after 4 hours of co-culture by flow cytometry. **(B–D)** Monocytes isolated from healthy donors (n = 4) previously infected 24 hours with **(*C*)** *burnetii* strains (50 MOI) or with *M. tuberculosis* (5 MOI) were co-cultured with Vγ9Vδ2 T cells expanded from healthy donor (E:T ratio of 1:1) in the presence of **(B)** anti-BTN2A (clone 7.48), **(C)** anti-BTN3A (clone 103.2) or **(D)** anti-BTN3A (clone 20.1) antibodies (10µg/ml). Vγ9Vδ2 T cell degranulation (%CD107ab+ cells) was assessed after 4 hours of co-culture by flow cytometry. **(E)** The cytotoxicity was assessed by flow cytometry as the percentage of Caspase 3/7^+^ cells in the target cell population after 4 hours of co-culture in presence of anti-BTN3A antibody (clone 20.1) (10µg/ml). Data were analyzed using a normality test and a parametric *t* test. Values represent mean ± standard deviation. *p < 0.05, **p < 0.01, ***p < 0.001 and ****p < 0.0001.

Since BTNs appeared to be over-expressed in monocytes following *C. burnetii* infection, we hypothesized that it could enhance the Vγ9Vδ2 T cell activation. After 4 hours of co-culture with *C. burnetii* infected monocytes, Vγ9Vδ2 T cell displayed enhanced degranulation as depicted by increased membrane expression of CD107, which also increased with the titer of bacteria used for monocytes infection (**Figure 4A**). We then investigated whether Vγ9Vδ2 T cell activation by *C. burnetii*-infected cells was dependent on BTNs by using anti-BTN3A antagonist (clone 103.2) (26) and anti-BTN2A antagonist (clone 7.48) (30) antibodies. Both antibodies led to significant inhibition of Vγ9Vδ2 T cell degranulation against cells infected with *C. burnetii* NMI or Guiana strains, or *M. tuberculosis* as positive control, suggesting that both BTNs are involved in Vγ9Vδ T cell activation in an infectious context (**Figures 4B, C**) as it was previously shown for malignant cells (30). Taken together, *C. burnetii* infection leads to Vγ9Vδ2 T cell activation in a BTN3A and BTN2A dependent manner.

We next hypothesized that Vγ9Vδ2 T cell activation towards *C. burnetii*-infected cells could be enhanced by an humanized BTN3A agonist antibody (clone 20.1) (26) that activates Vγ9Vδ2 T cells. As illustrated in the **Figure 4D**, we observed that the BTN3A activating antibody leads to increased expression of CD107 (**Figure 4D**) and the cytotoxic activity (**Figure 4E**) of Vγ9Vδ2 T cells towards *C. burnetii* infected monocytes as observed for *M. tuberculosis* after 4 hours of co-culture. A similar effect was observed for all *C. burnetii* strains, to the same extent as *M. tuberculosis*, suggesting that the 20.1 antibody can induce Vγ9Vδ2 T cell activation even towards virulent bacteria. These data show that targeting Vγ9Vδ2 T cells with the 20.1 antibody leads to the activation of their cytotoxicity against *C. burnetii*-infected cells.

### Anti-BTN3A Agonist Antibody Increases Antimicrobial Activity of Vγ9Vδ2 T Cells

**Figure 5:**
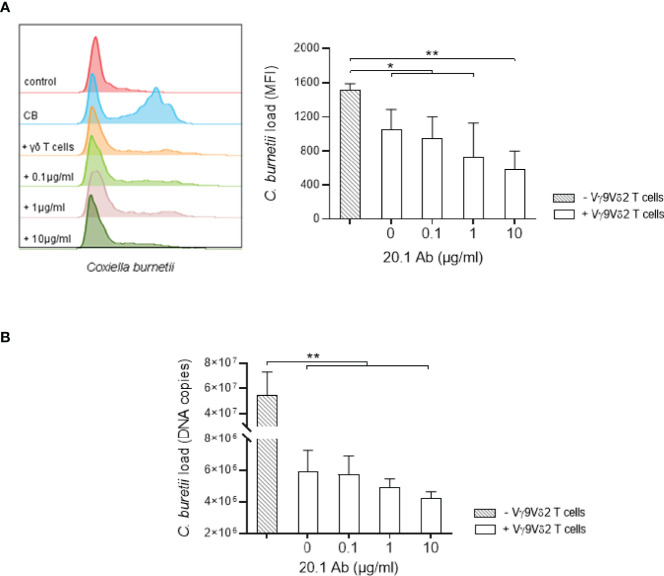
Anti-BTN3A agonist antibody increases antimicrobial activity of Vγ9Vδ2 T cells towards *C. burnetii* infected monocytes. **(A, B)** Monocytes isolated from healthy donors (n = 4) previously infected 24 hours with *C. burnetii* NM1 (50 MOI) were co-cultured with autologous Vγ9Vδ2 T cells (E:T ratio of 1:1) in the presence of anti-BTN3A antibody (clone 20.1) (0-10 µg/ml). After 4 hours of co-culture, *C. burnetii* load was measured by **(A)** flow cytometry and **(B)** qPCR. Data were analyzed using a normality test and a parametric *t* test. Values represent mean ± standard deviation. *p < 0.05, **p < 0.01.

Since the anti-BTN3A agonist antibody (clone 20.1) increases Vγ9Vδ2 T cell activation, we wondered whether it was able to boost their antimicrobial activity. For this purpose, monocytes were infected with *C. burnetii* NM1 for 24 hours and then co-cultured with Vγ9Vδ2 T cells for 4 hours in presence of 20.1 antibody (0, 0.1, 1 or 10 µg/ml) and the bacterial load was measured by flow cytometry and qRT-PCR. First, Vγ9Vδ2 T cells lead to a significant reduction of *C. burnetii* load from 5.10^7^ to 6.10^6^ in monocytes in the presence of Vγ9Vδ2 T lymphocytes (p=0.0021) (**Figures 5A, B**), as observed for *M. tuberculosis* infection (**Supplementary Figure 3**). BTN3A activating antibody resulted in a dose-dependent decrease in *C. burnetii* load in monocytes, reaching from 6.10^6^ to 4.2.10^6^ (0 *vs.* 10 µg/ml, p=0.0501) (**Figures 5A, B**). This effect is similar to that observed in the case of *M. tuberculosis*, where the 20.1 antibody resulted in a decrease in the bacterial load in monocytes (0 *vs.* 10 µg/ml, p=0.0158) (**Supplementary Figure 3**). Altogether, BTN3A activating antibody increases the antimicrobial activity of Vγ9Vδ2 T lymphocytes against monocytes infected with *C. burnetii*.

### Anti-BTN3A Agonist Antibody Increases the Secretion of Cytokines and Cytotoxic Molecules by Vγ9Vδ2 T Cells

**Figure 6:**
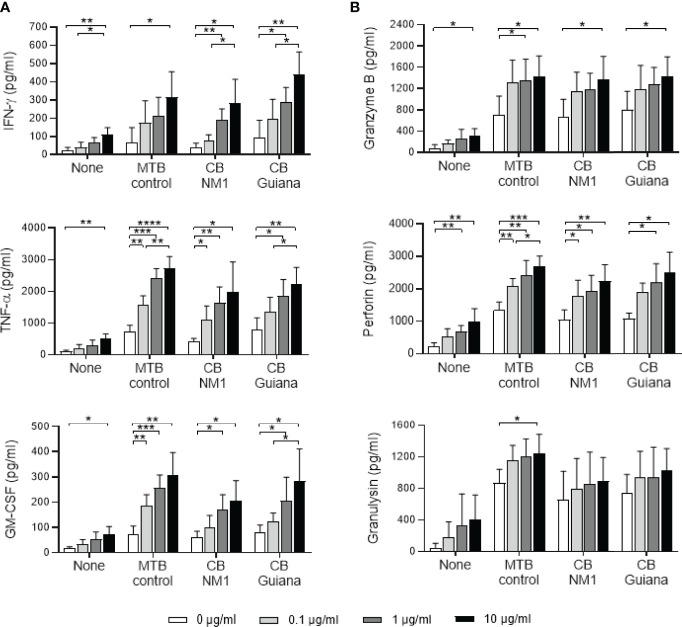
Anti-BTN3A agonist antibody increases the secretion of cytokines and cytotoxic molecules in Vγ9Vδ2 T cell/infected-monocyte co-cultures. Monocytes isolated from healthy donors (n = 4) previously infected 24 hours with *C. burnetii* NM1 (50 MOI) or with *M. tuberculosis* (5 MOI) were co-cultured with autologous Vγ9Vδ2 T cells (E:T ratio of 1:1) in the presence of anti-BTN3A antibody (clone 20.1) (0-10 µg/ml). After 4 hours of co-culture, the culture supernatants were analyzed for the presence of cytokines (**A**, left panel) and cytotoxic molecules (**B**, right panel) by ELISA assay. Data were analyzed using a normality test and a parametric *t* test. Values represent mean ± standard deviation. *p < 0.05, **p < 0.01, ***p < 0.001 and ****p < 0.0001.

Since treatment with the anti-BTN3A agonist antibody leads to bacterial load reduction, we investigated whether this could be related to the secretion of cytokines and cytotoxic molecules, which are strongly produced by activated Vγ9Vδ2 T cells (17–21). Indeed, treatment of Vγ9Vδ2 T cell/*C. burnetii-*infected monocyte co-cultures with the 20.1 mAb increased TFN-α, IFN-γ and GM-CSF secretion in a dose-dependent manner (**Figure 6A**, left panel). Moreover, a significant difference was observed between the 0.1 and 10 µg/ml doses for IFN-γ, TFN-α and GM-CSF secretion (p=0.0260, p=0.0443 and p=0.0265, respectively), in the case of infection with *C. burnetii* Guiana. Regarding cytotoxic molecules, granzyme B and perforin secretion were significantly increased in presence of 10 µg/ml of 20.1 mAb in the case of monocytes infected with *C. burnetii* NM1 and Guiana, *M. tuberculosis* and uninfected monocytes (**Figure 6B**, right panel). On the other hand, the 20.1 mAb showed a less pronounced effect on granulysin secretion, with a significant difference only in the case of *M. tuberculosis* infection (0 *vs.* 10 µg/ml, p=0.0488). It can also be noted that the levels of granulysin appeared to be higher in the case of *M. tuberculosis* infection than with *C. burnetii*. Overall, the presence of the BTN3A activating antibody increases the secretion of cytokines and cytotoxic molecules, both produced by the activated Vγ9Vδ2 T cells.

## Discussion

An alteration of circulating Vγ9Vδ2 T cells has been observed in Q fever patients (10). During acute phase of the disease, the proportion of Vγ9Vδ2 T cells is significantly increased in patients (16% *vs.* 4% in healthy donors) (10), indicating the involvement of these cells in the acute immune response to *C. burnetii*. Since human Vγ9Vδ2 T cell responses are triggered *via* an interaction with the BTN2A1/BTN3A1 complex, we first assessed whether their expression was modulated following *C. burnetii* infection. We found that *in vitro* infection of monocytes with *C. burnetii* induced a significant increase in the transcriptomic and plasma membrane expression of these two BTNs. This increase is similar to that observed with *M. tuberculosis* and between *C. burnetii* strains, suggesting that the aggressiveness of the bacteria appears to have limited impact on BTN expression. Similarly, increased expression of these two BTNs has recently been described in red blood cells infected by *Plasmodium falciparum* (42). Our team has recently shown higher expression of BTN3A, but not BTN2A, following SARS-CoV-2 infection of myeloid cells and lung cell lines (submitted manuscript). This may suggest different mechanisms depending on the pathogen.

Using CRISPR-Cas9 gene inactivation in the THP-1 cell line, we found that BTN3A and BTN2A are not directly involved in the infection process of cells by *C. burnetii* but play a role in the cellular immune response to infection. Indeed, THP-1 cells inactivated for BTN3A or BTN2A show a repressed inflammatory response following *C. burnetii* infection, with a significant decrease in *TNF* and *IL1B* gene expression. These results suggest that higher expression of these two molecules on monocytes could favor responses to *C. burnetii* infection.

The fact that both BTN3A and BTN2A, essential for Vγ9Vδ2 T cell activation, are more expressed following *C. burnetii* infection could enhance their activation and antibacterial activity. Using a Vγ9Vδ2 T cell/infected monocyte co-culture model, we observed that monocytes infected with *C. burnetii* strains of different aggressiveness resulted in similar degranulation of Vγ9Vδ2 T cells. Several studies have confirmed that the activation of Vγ9Vδ2 T cells is dependent on BTN3A during infections. Indeed, the anti-BTN3A antagonist antibody 103.2 was able to inhibit the degranulation of Vγ9Vδ2 T cells when they were co-cultured with cells infected with *M. bovis* (BCG), *L. monocytogenes*, *P. falciparum* or Epstein-Barr virus (23, 42–44). In our study, similar results are obtained with the 103.2 antibody but are also observed with an anti-BTN2A antagonist antibody (clone 7.48), underlining the importance of these two BTNs in the activation of Vγ9Vδ2 T cells.

Next, we evaluated the effect of BTN3A on antibacterial activity. For this purpose, we used the anti-BTN3A agonist antibody 20.1 to treat Vγ9Vδ2 T cell/*C. burnetii*-infected monocyte co-cultures. Our results show that the 20.1 mAb increases the antibacterial activity of Vγ9Vδ2 T cells leading to a decreased intracellular load of *C. burnetii*. In our study, Vγ9Vδ2 T cells, whose cytotoxic activity is enhanced by the 20.1 mAb, were both able to kill *C. burnetii*-infected monocytes through the production of lytic granules (granulysin, perforin, granzymes) and at the same time produce large amounts of IFN-γ and TFN-α. These cytokines play an essential role in protection against intracellular bacteria by activating the antimicrobial machinery of phagocytes. Indeed, IFN-γ induces *C. burnetii* killing by promoting apoptosis of infected monocytes (36, 45), and TNF-α shows an essential role in the control of *C. burnetii* infection like for other pathogens including *M. tuberculosis* or *L. monocytogenes* (46, 47). These data extend previous studies as human Vγ9Vδ2 T cells have already been shown to effectively kill intracellular pathogens, such as *M. tuberculosis*, *L. monocytogenes* and *B. suis*, through the secretion of IFN-γ, TNF-α and cytotoxic molecules such as granzymes, perforin and granulysin (17–21, 48). Some studies have also reported that NKG2D contributed to the anti-infective activity of Vγ9Vδ2 T cells against *Brucella* sp. and *M. tuberculosis* (49, 50). In contrast, in other studies on *M. tuberculosis* or *L. monocytogenes*, NKG2D was not involved (20, 43). These discrepancies may be due to the different expression of NKG2D ligands between infections and between cell populations. Diverse functions of NKG2D ligands could have an impact on the anti-infective activity of Vγ9Vδ2 T cells.

Our data suggest that targeting Vγ9Vδ2 T cells to activate their cytotoxic functions may be considered a promising strategy for the treatment wide range of pathogens like for *C. burnetii*. Indeed, alterations in the phenotype and/or functions of Vγ9Vδ2 T cells have been reported in several infections usually caused by intracellular pathogens. For example, in patients with active tuberculosis, a progressive loss of effector function of circulating Vγ9Vδ2 T cells has been reported, leading to decreased IFN-γ production and granulysin expression (51, 52). This alteration was correlated with disease progression (53, 54), suggesting that a high level of bacteria can lead to chronic stimulation of Vγ9Vδ2 T cells that would result in their apoptosis and/or senescence. Targeting Vγ9Vδ2 T cells in the context of persistent infections could therefore be an attractive strategy. Future phenotypic and functional analyses of Vγ9Vδ2 T cells from patients with Q fever will allow to determine whether their capacity is altered.

Recently, a novel approach has been developed to expand and activate Vγ9Vδ2 T cells besides pAgs. This strategy is based on the development of a new class of molecules called immunoantibiotics, in particular the inhibitor IspH (55). IspH, an enzyme of the isoprenoid synthesis pathway, is essential for the survival of most Gram-negative bacteria and the absence of IspH causes an accumulation of its substrate HMBPP, which in turn activates Vγ9Vδ2 T cells. Another approach would be to target specifically the ligands expressed on the surface of stressed infected cells, such as BTN3A, which will vehicle activation and cytotoxicity of Vγ9Vδ2 T cells (56). This is the case in a trial in cancer patients where the approach is to activate Vγ9Vδ2 T cells by targeting BTN3A (NCT04243499, ImCheck Therapeutics, Marseille, France) (57, 58).

In addition, we have also explored the effect of the 20.1 mAb in the case of SARS-CoV-2 infection. By activating the Vγ9Vδ2 T cells, 20.1 mAb may affect intracellular SARS-CoV-2 replication *in vitro* in infected cells (submitted manuscript). Future studies should be conducted to elucidate the detailed mechanisms of protective Vγ9Vδ2 T cell activation and how precisely BTN3A is involved in infections. These results highlight that the BTN3A agonist antibody could represent powerful therapeutic tool in infections to overcome the imbalances in immune responses observed in some patients and open new perspectives in Vγ9Vδ2 T-cell-based immunotherapies in infectious diseases.

In summary, this study provided further insight into the role of Vγ9Vδ2 T cells in infections with intracellular bacteria. We demonstrated that *C. burnetii* infection results in modulation of BTN3A and BTN2A co-receptor expression, allowing activation of Vγ9Vδ2 T cells. We report for the first time the role of a BTN3A agonist antibody in the control of intracellular bacterial infection. The latter boosts the cytotoxic functions of Vγ9Vδ2 T cells *in vitro* such as their degranulation, the production of TNF-α and IFN-γ, and killing activity leading to a better clearance of *C. burnetii* load of infected target cells. These results may facilitate new approaches to the treatment of persistent bacterial infections by enhancing Vγ9Vδ2 T cell responses in presence of infected cells.

## Data Availability Statement

The original contributions presented in the study are included in the article/**Supplementary Material**. Further inquiries can be directed to the corresponding author.

## Ethics Statement

A convention No.7828 was established between our laboratory and the Etablissement Français du Sang (Marseille, France). The patients/participants provided their written informed consent to participate in this study.

## Author Contributions

LG, MG, and MF performed the experiments and analyzed the data. SM, CC, EF, L.M, J-LM and DO supervised the work. LG, SM, J-LM, and DO participated in the writing of the paper. All the authors read and approved the final manuscript.

## Funding

LG was supported by a Cifre fellowship from ImCheck Therapeutics.

## Conflict of Interest

DO is cofounder and shareholder of Imcheck Therapeutics, Emergence Therapeutics, Alderaan Biotechnology and Stealth IO. CC, EF, MG, MF, and LM are employees and shareholders of Imcheck Therapeutics.

The remaining authors declare that the research was conducted in the absence of any commercial or financial relationships that could be construed as a potential conflict of interest.The authors declare that this study received funding from ImCheck Therapeutics. The funder had the following involvement in the study: study design, collection, analysis, interpretation of data and the writing of this article.

## Publisher’s Note

All claims expressed in this article are solely those of the authors and do not necessarily represent those of their affiliated organizations, or those of the publisher, the editors and the reviewers. Any product that may be evaluated in this article, or claim that may be made by its manufacturer, is not guaranteed or endorsed by the publisher.

## Supplementary Material

Supplementary Figure 1. Phenotype confirming gene invalidation in THP-1 cell line. CRISPR-Cas9-mediated inactivation of BTN3A1/3A2/3A3 or BTN2A1/2A2 isoforms was performed in THP-1 cell lines. (A) The expression level of BTN3A was assessed by flow cytometry. Data were collected on a BD Canto II instrument (BD Biosciences). (B) The expression level of BTN2A was assessed by flow cytometry. Data were collected on a CytoFLEX S instrument (Beckman Coulter). All data were analyzed with FlowJo software (FlowJo v10.6.2).

Supplementary Figure 2. Manual gating for Vγ9Vδ2 T cell functional assays. (A) Monocytes were co-cultured with Vγ9Vδ2 T cells at effector-to-target (E:T) ratio of 1:1 and fluorochrome-labeled CD107a and CD107b. Phorbol 12-myristate 13-acetate (PMA, 20 ng/mL) with ionomycine (1 µg/mL) were used as positive control for Vγ9Vδ2 T cell activation. After 4 hours, cells were harvested and stained with fluorochrome-labeled TCR-specific mAbs and a viability marker. The degranulation was evaluated by flow cytometry as the percentage CD107a/b+ cells in the γδ T cell population. (B) Monocytes were labeled with 10 µM Cell Proliferation Dye eFluor® 670 and then co-cultured with Vγ9Vδ2 T cells at E:T ratio of 1:1. After 4 hours, cells were stained with CellEvent Caspase-3/7 Green to identify dead cells. The cytotoxicity was assessed by flow cytometry as the percentage of Caspase 3/7+ cells in the target cell population.

Supplementary Figure 3. Anti-BTN3A agonist antibody increases antimicrobial activity of Vγ9Vδ2 T cells towards M. tuberculosis infected monocytes. Monocytes isolated from healthy donors (n=4) previously infected 24 hours with M. tuberculosis (5 MOI) were co-cultured with autologous Vγ9Vδ2 T cells (E:T ratio of 1:1) in the presence of anti-BTN3A antibody (clone 20.1) (0-10 µg/ml). After 4 hours of co-culture, M. tuberculosis load was measured by qPCR. Data were analyzed using a normality test and a Mann-Whitney U test. Values represent mean ± standard deviation. *p < 0.05, **p < 0.01 and ***p < 0.001.
