# Supplementary figures and images for: BTN3A Targeting Vγ9Vδ2 T Cells Antimicrobial Activity Against Coxiella burnetii-Infected Cells
Source: Front Immunol. 2022 Jun 27;13:915244. doi: 10.3389/fimmu.2022.915244 (PMC9272908; doi:10.3389/fimmu.2022.915244)

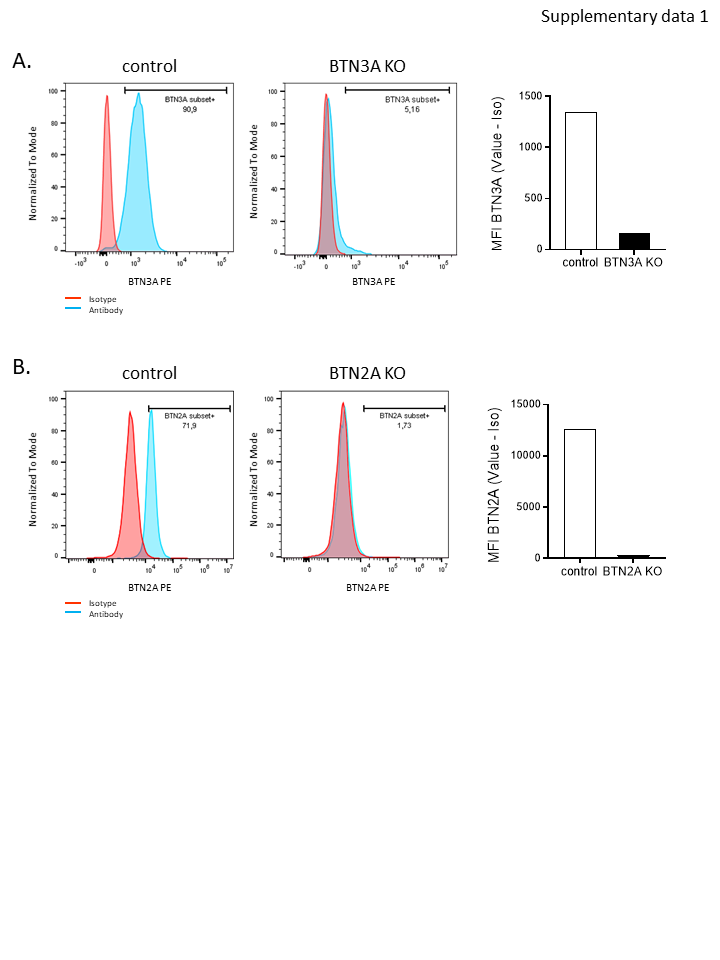

Supplement: Supplementary Figure 1. — Phenotype confirming gene invalidation in THP-1 cell line. CRISPR-Cas9-mediated inactivation of BTN3A1/3A2/3A3 or BTN2A1/2A2 isoforms was performed in THP-1 cell lines. (A) The expression level of BTN3A was assessed by flow cytometry. Data were collected on a BD Canto II instrument (BD Biosciences). (B) The expression level of BTN2A was assessed by flow cytometry. Data were collected on a CytoFLEX S instrument (Beckman Coulter). All data were analyzed with FlowJo software (FlowJo v10.6.2). [file Image_1.tif]

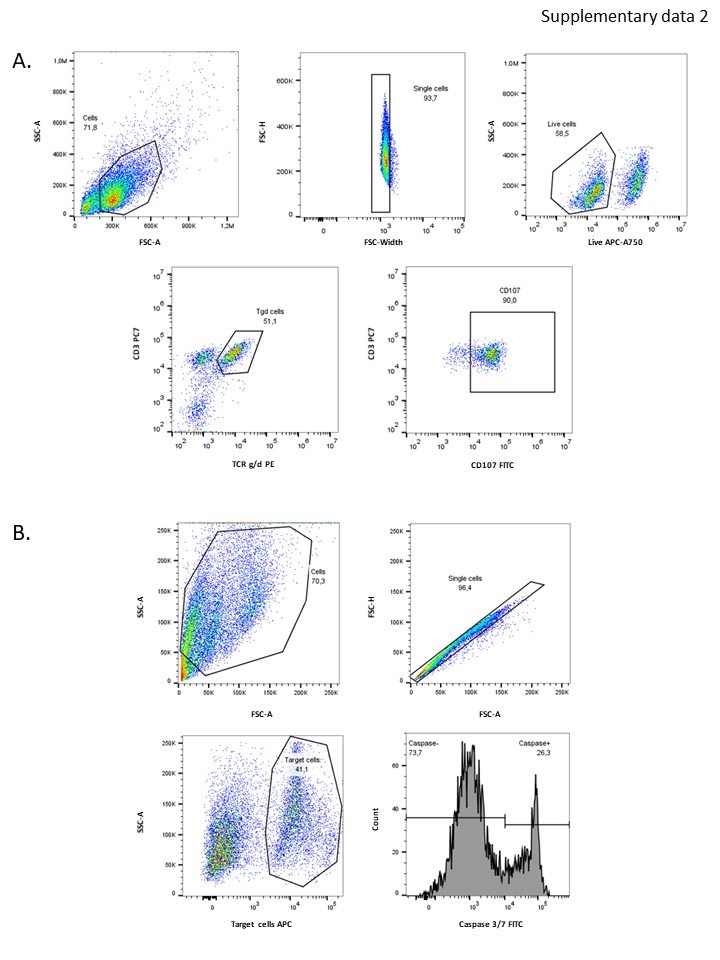

Supplement: Supplementary Figure 2. — Manual gating for Vγ9Vδ2 T cell functional assays. (A) Monocytes were co-cultured with Vγ9Vδ2 T cells at effector-to-target (E:T) ratio of 1:1 and fluorochrome-labeled CD107a and CD107b. Phorbol 12-myristate 13-acetate (PMA, 20 ng/mL) with ionomycine (1 µg/mL) were used as positive control for Vγ9Vδ2 T cell activation. After 4 hours, cells were harvested and stained with fluorochrome-labeled TCR-specific mAbs and a viability marker. The degranulation was evaluated by flow cytometry as the percentage CD107a/b+ cells in the γδ T cell population. (B) Monocytes were labeled with 10 µM Cell Proliferation Dye eFluor® 670 and then co-cultured with Vγ9Vδ2 T cells at E:T ratio of 1:1. After 4 hours, cells were stained with CellEvent Caspase-3/7 Green to identify dead cells. The cytotoxicity was assessed by flow cytometry as the percentage of Caspase 3/7+ cells in the target cell population. [file Image_2.tif]
